# MIF Increases sFLT1 Expression in Early Uncomplicated Pregnancy and Preeclampsia

**DOI:** 10.3390/ijms241210050

**Published:** 2023-06-13

**Authors:** Qing Yong, Kyra L. Dijkstra, Carin van der Keur, Jan A. Bruijn, Michael Eikmans, Hans J. Baelde

**Affiliations:** 1 Department of Pathology, Leiden University Medical Center, 2333 ZA Leiden, The Netherlands; q.yong@lumc.nl (Q.Y.);; 2 Department of Immunology, Leiden University Medical Center, 2333 ZA Leiden, The Netherlands

**Keywords:** macrophage migration inhibitory factor, soluble fms-like tyrosine kinase-1, inflammation, preeclampsia

## Abstract

Insufficient immune tolerance during pregnancy is associated with pathological conditions such as preeclampsia (PE). Soluble fms-like tyrosine kinase-1 (sFLT1), which exerts a role in the late stage of PE, has shown its beneficial anti-inflammatory effects in inflammation-associated diseases. Macrophage migration inhibitory factor (MIF) was reported to upregulate sFLT1 production in experimental congenital diaphragmatic hernia. However, the placental sFLT1 expression in early uncomplicated pregnancy and whether MIF can regulate sFLT1 expression in uncomplicated and preeclamptic pregnancy are unclear. We collected first-trimester placentas and term placentas from uncomplicated and preeclamptic pregnancies to investigate sFLT1 and MIF expression in vivo. Primary cytotrophoblasts (CTBs) and a human trophoblast cell line (Bewo) were used to study the regulation of MIF on sFLT1 expression in vitro. In placentas from first-trimester pregnancy, we observed a high expression of sFLT1, specifically in extravillous trophoblasts (EVTs) and syncytiotrophoblast (STB) cells. *MIF* mRNA levels strongly correlated with *sFLT1* expression in term placentas from preeclamptic pregnancies. In in vitro experiments, *sFLT1* and *MIF* levels increased significantly in CTBs during their differentiation to EVTs and STBs, and MIF inhibitor (ISO-1) significantly reduced *sFLT1* expression in a dose-dependent manner during this process. *sFLT1* showed significant upregulation with increasing doses of MIF in Bewo cells. Our results show that sFLT1 is highly expressed at the maternal–fetal interface during early pregnancy and that MIF can increase sFLT1 expression in early uncomplicated pregnancy and PE, which suggests that sFLT1 plays an essential role in the modulation of inflammation in pregnancy.

## 1. Introduction

It is well known that appropriate placentation in the first trimester is essential for pregnancy maintenance, and that defective placental development can lead to pregnancy complications such as miscarriage, fetal growth restriction, and preeclampsia (PE) [1,2]. Upon implantation, cytotrophoblasts (CTBs) undergo proliferation and differentiate into either multinucleated syncytiotrophoblast (STB) or invasive extravillous trophoblasts (EVTs). The STB serves as the site of gas and nutrient exchange between the mother and the fetus. The EVTs are composed of two subtypes of cells, one of which invades deeply into the maternal decidua, termed interstitial EVT, and the endovascular EVTs that remodel maternal spiral vessels, establishing maternal blood flow into the intervillous space [3,4].

As the only fetal-derived cells carrying paternal antigens, both EVTs and STB play a core role in mediating maternal tolerance. They come into direct contact with maternal immune cells at the maternal–fetal interface and contribute to the unique immunotolerant microenvironment via cell–cell direct interaction and through actions of chemokines and cytokines [5]. Immune tolerance is necessary to protect the fetal cells against attack from the maternal immune system and ensure a successful pregnancy [6]. Inadequate or insufficient anti-inflammatory responses followed by excessive inflammation is thought to be an important trigger for pathological conditions such as PE [7].

Increased levels of soluble fms-like tyrosine kinase-1 (sFLT1) reflect a key factor driving PE at the late stage as an antagonist of vascular endothelial growth factor (VEGF) and placental growth factor (PIGF) [8,9,10]. Another study suggested that in PE, placental sFLT1 is upregulated as a protective response to an increase in endometrial VEGF levels, which is associated with placental deformities and pregnancy loss [11]. In line with this, several studies have shown that sFLT1 has beneficial anti-inflammatory properties in inflammation-associated diseases, such as psoriasis and type 1 diabetes [12,13].

Despite great interest in the potential beneficial immuno-modulatory properties of sFLT1 in pregnancy and its pathogenic role in the late stage of PE, the expression and regulation of sFLT1 are not fully understood. Hypoxia leads to the upregulation of sFLT1 expression [14]. However, in vivo and in vitro studies also suggest that inflammation-associated factors such as macrophage migration inhibitory factor (MIF) increase sFLT1 expression [15,16,17]. MIF is a versatile cytokine acting as a central regulator of different physiological processes including cell proliferation and differentiation, angiogenic biological activities, and innate immune response [18]. It can be produced by immune cells, endothelial, epithelial, and many other cell types [19]. MIF is abundantly expressed at the maternal–fetal interface and is proposed to have a role in establishing and maintaining a healthy pregnancy [20]. MIF has also been associated with some placental pathological conditions, including infection and PE [20]. Additionally, it has been shown that MIF increases sFLT1 production in experimental congenital diaphragmatic hernia [21]. Therefore, we hypothesized that placental sFLT1 has high expression at the maternal–fetal interface during pregnancy and that MIF is involved in the regulation of increased placental sFLT1 production during normal and preeclamptic pregnancy.

## 2. Results

### 2.1. High Expression of sFLT1 in EVTs and STB in Placentas during First Trimester

**Figure 1:**
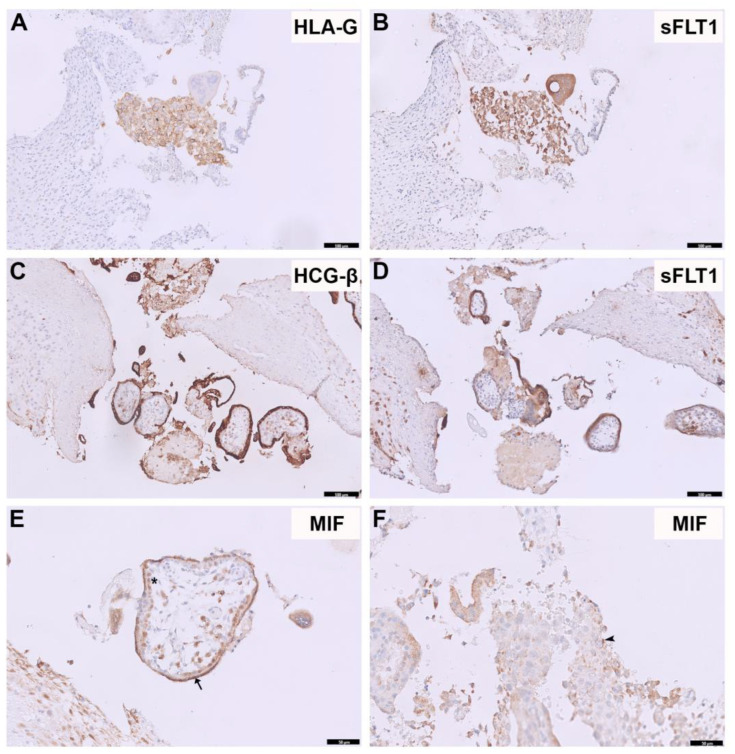
sFLT1 and MIF expression in placentas during first trimester. HLA-G and HCG-β were stained as markers for EVTs and STBs, respectively. Representative images of HLA-G (**A**) and sFLT1 (**B**) staining in the same area; HCG-β (**C**) and sFLT1 (**D**) in the same area show sFLT1 expression in both EVTs and STB. Representative MIF staining (**E**,**F**) in STBs (arrow), CTBs (star), and EVTs (arrowhead). The scale bars of (**A**–**D**) represent 100 µm; the scale bars of (**E**,**F**) represent 50 µm.

To investigate the expression of sFLT1 and MIF at the maternal–fetal interface in early pregnancy, we performed immunohistochemistry (IHC) on placentas during the first trimester. HLA-G and HCG-β were stained as markers of EVTs and STB, respectively. Representative images are shown in Figure 1. There was a high expression of sFLT1 in EVTs (Figure 1B) and STB (Figure 1D), while MIF staining can be seen in CTBs, EVTs, and STB (Figure 1E,F).

### 2.2. sFLT1 and MIF Expression Increases upon Human Primary Cytotrophoblast Differentiation into EVTs and STBs

**Figure 2:**
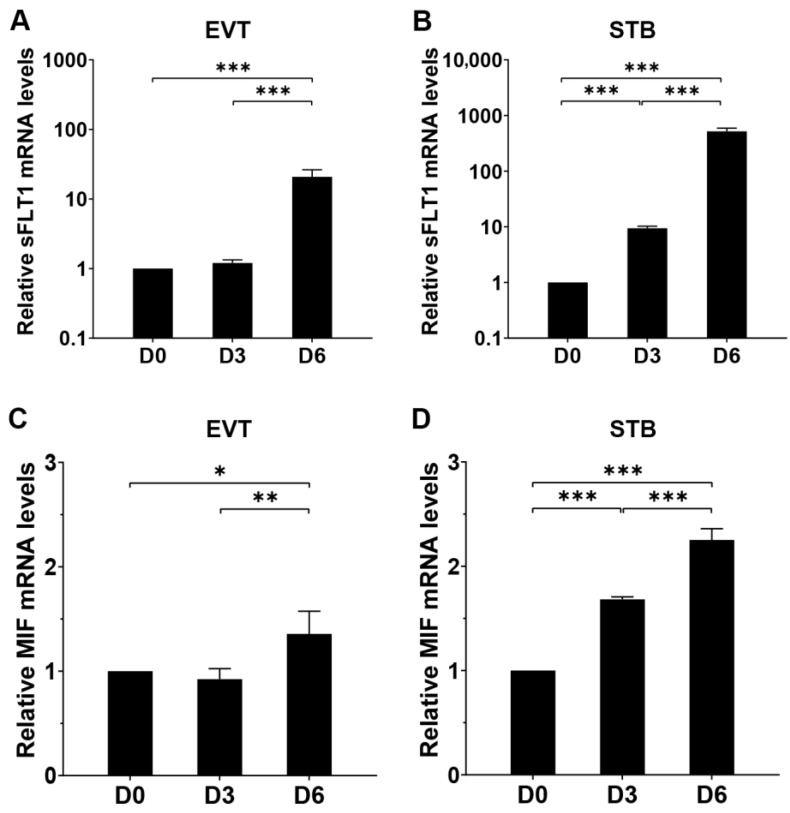
*sFLT1* and *MIF* expression during differentiation of human primary CTBs into EVTs and STB. CTBs were incubated with EVT or STB medium for 6 days; qPCR was used to measure *sFLT1* and *MIF* expression on day 0 (CTBs), 3, and 6. Data were expressed relative to CTBs (day 0). Results were corrected for the housekeeping gene *GAPDH*. Summary data are presented as means ± SD from three independent experiments. The mRNA levels of *sFLT1* (**A**,**B**) and *MIF* (**C**,**D**) during differentiation of CTBs into EVTs (**A**,**C**) and STBs (**B**,**D**). One-way ANOVA on log-transformed gene expression was performed, * *p* < 0.05, ** *p* < 0.01, *** *p* < 0.001.

Next, to confirm MIF and sFLT1 expression in vitro, human primary CTBs were isolated from first-trimester placentas and differentiated into EVTs or STBs. Quantitative PCR (qPCR) was used to measure *sFLT1* and *MIF* mRNA levels on day 0 (undifferentiated CTBs) and 3 and 6 days after differentiation. Upon CTB differentiation into EVTs, *sFLT1* mRNA expression had a 1.2-fold increase (*p* = 0.456) on day 3 and increased 21-fold on day 6 (*p* < 0.001) compared to day 0 (CTBs) (Figure 2A). The mRNA level of *MIF* was similar on day 3 and rose by 1.4 fold (*p* < 0.01) on day 6 (Figure 2C) compared to CTBs. When CTBs were differentiated to STB, *sFLT1* mRNA levels increased substantially by 9 fold (*p* < 0.001) on day 3 and 518 fold (*p* < 0.001) on day 6 (Figure 2B). For *MIF* mRNA expression, it increased 1.7-fold (*p* < 0.001) on day 3 and 2.3 fold (*p* < 0.001) on day 6 (Figure 2D).

### 2.3. MIF Inhibitor Reduces sFLT1 Expression during Differentiation of CTBs into EVTs and STB 

**Figure 3:**
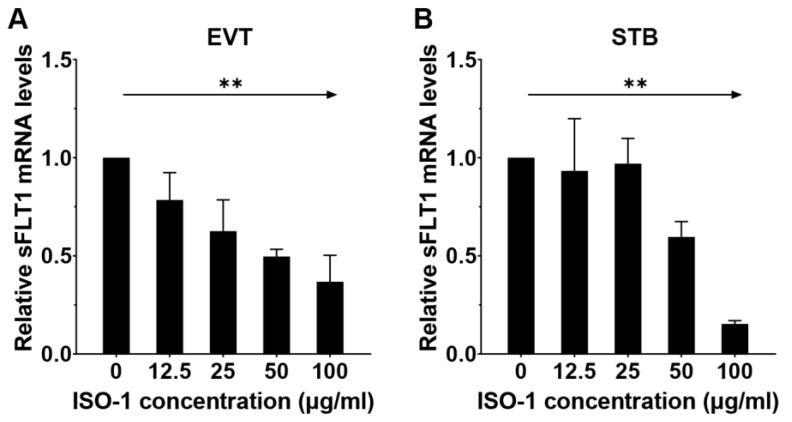
MIF inhibitor reduces sFLT1 expression during differentiation of CTBs into EVTs and STB. CTBs were incubated with EVT or STB medium for 6 days. Different concentrations (12.5 µg/mL, 25 µg/mL, 50 µg/mL, and 100 µg/mL) of MIF inhibitor (ISO-1) were added to the medium from day 3 onwards. After that, *sFLT1* mRNA expression was measured on D6 using qPCR. As a control, cells were cultured in differentiation medium without ISO-1. Data were expressed relative to their own control without ISO-1. *sFLT1* mRNA levels during differentiation of CTBs into EVTs (**A**) and STB (**B**) without or with an increased dose of ISO-1. Linear regression analysis on log-transformed ISO-1 concentration and *sFLT1* mRNA expression, taking experiments into account. ** *p* < 0.01.

To study whether MIF can regulate sFLT1 expression during differentiation from CTBs to EVTs or STB, we incubated CTBs in EVT or STB medium with or without an increasing dose of MIF inhibitor ISO-1 (12.5 µg/mL, 25 µg/mL, 50 µg/mL, and 100 µg/mL). We found a significant dose-dependent decrease in *sFLT1* mRNA expression after incubation with MIF inhibitor ISO-1. During differentiation of CTBs into EVTs with ISO-1, the expression of *sFLT1* at mRNA level reduced by 0.4 fold with every 10-fold increase in ISO-1 concentration (Figure 3A, *p* < 0.01), and *sFLT1* mRNA levels showed a 0.14-fold decrease (Figure 3B, *p* < 0.01) when STB was differentiated from CTBs in the presence of ISO-1.

### 2.4. MIF Increases sFLT1 Expression in a Human Trophoblast Cell Line

**Figure 4:**
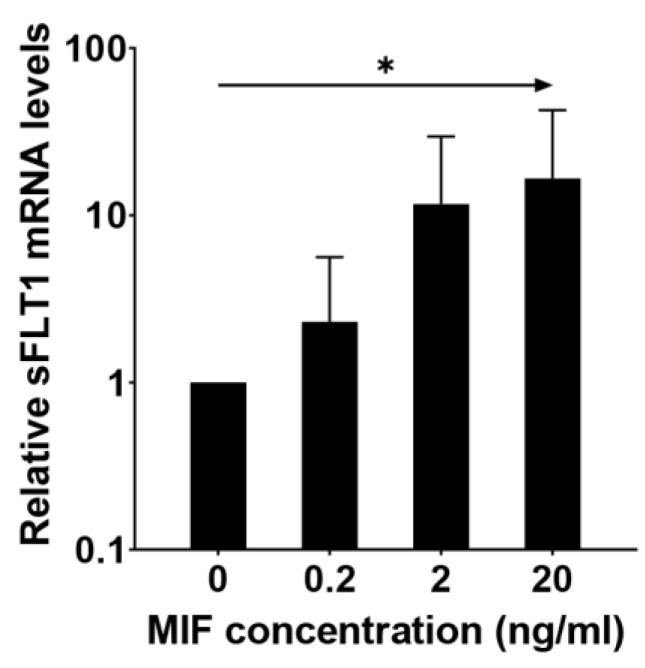
MIF increases sFLT1 expression in a human trophoblast cell line. Bewo cells were incubated without or with 0.2 ng/mL, 2 ng/mL, and 20 ng/mL of MIF for 24 h, after which *sFLT1* mRNA levels were measured. Linear regression analysis was performed on log-transformed MIF concentration and *sFLT1* mRNA expression. * *p* < 0.05.

As sFLT1 already increased in expression during differentiation, we wanted to analyze the regulatory properties of MIF on sFLT1 expression in human trophoblasts. Therefore, we cultured Bewo cells, a widely used human trophoblast cell line, and stimulated these cells with different concentrations of MIF (0.2 ng/mL, 2 ng/mL, and 20 ng/mL). With each 10-fold increase in MIF concentration, the expression of *sFLT1* showed a 2.3-fold elevation (Figure 4, *p* < 0.05).

### 2.5. MIF Expression Correlates with sFLT1 Expression in Placentas from Preeclamptic Women

**Figure 5:**
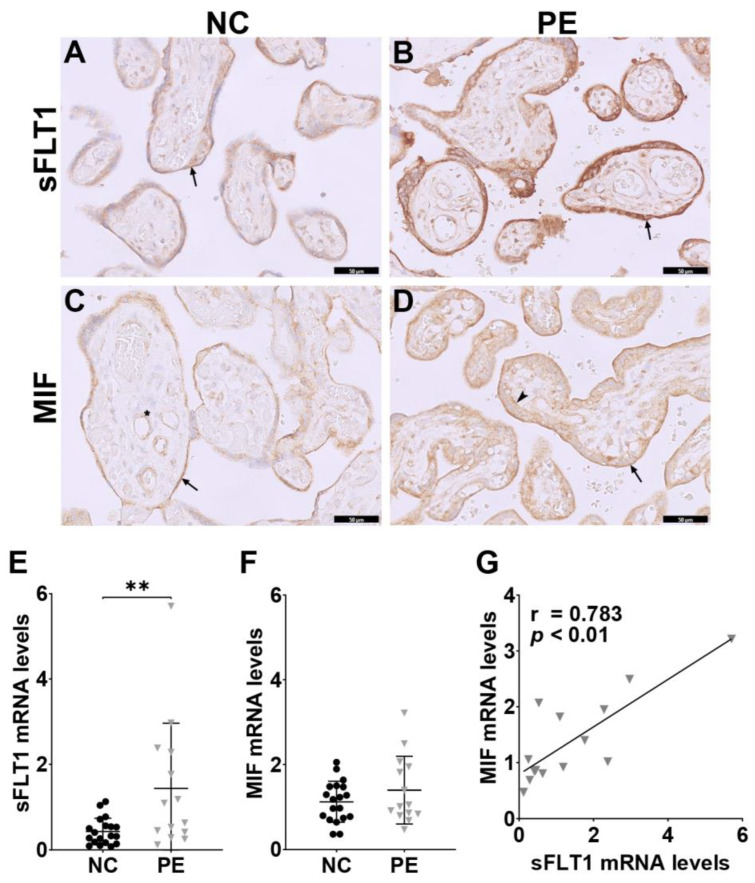
Correlation between *sFLT1* mRNA levels and *MIF* mRNA levels in PE. Representative images of the sFLT1 (**A**,**B**) and MIF (**C**,**D**) staining in placental tissue from normal pregnancy (NC) and from patient with preeclampsia (PE). sFLT1 is expressed mainly in STB (arrows), MIF is present in STB (arrows), CTBs (arrowhead), and fetal endothelial cells (star). The scale bars represent 50 µm. *sFLT1* (**E**) and *MIF* (**F**) mRNA levels were measured in two groups using qPCR. Independent *t*-test on log-transformed mRNA expression was performed. ** *p* < 0.01. (**G**) Correlation plots show a significant correlation between *MIF* and *sFLT1* mRNA expression in the PE group. Bivariate Pearson correlation using log-transformed values was used.

We first performed IHC for sFLT1 and MIF on normal control (NC) and preeclamptic (PE) placentas. Representative images of the sFLT1 and MIF staining of an NC and PE are shown in Figure 5A–D. MIF was mainly present in STB, CTBs, and fetal endothelial cells (Figure 5C,D), while sFLT1 was expressed prominently in STB (Figure 5A,B). We scored MIF expression semi-quantitatively and did not find a significant difference between the NC (N = 19) and PE (n = 14) groups (*p* = 0.541).

Next, we measured *sFLT1* and *MIF* mRNA levels in placentas using qPCR. The expression of *sFLT1* mRNA increased significantly in the PE group compared to the NC group (Figure 5E, *p* < 0.01). *MIF* mRNA expression had a mild elevation in PE, but this increase was not significant (Figure 5F, *p* = 0.335).

Finally, to investigate the relationship between MIF and sFLT1 expression in PE, we correlated placental *sFLT1* with *MIF* mRNA levels. Within the PE group, there was a significant positive correlation between *MIF* mRNA and *sFLT1* mRNA levels (Figure 5G, *p* < 0.01).

## 3. Discussion

Here, we showed that in early uncomplicated pregnancy, sFLT1 is highly expressed in EVTs and STB, and that placental sFLT1 increases significantly in PE compared to uncomplicated term pregnancy. Moreover, we showed that MIF increases sFLT1 production in cultured STB and that MIF inhibitor ISO-1 reduces sFLT1 expression during differentiation of CTBs into EVTs and STB in vitro. Furthermore, we showed a strong correlation between *sFLT1* mRNA and *MIF* mRNA in term placental tissue of patients with PE. Together, our findings suggest high placental sFLT1 expression at the maternal–fetal interface during early pregnancy and MIF as an important mediator in regulating sFLT1 expression in early uncomplicated pregnancy and PE.

The finding that placental sFLT1 expression has a significant increase in PE is in line with the current literature. Abundant studies have described that placental sFLT1 mRNA and protein levels increase significantly in PE compared to normotensive term pregnancy [8,22,23]. Additionally, the study by Buurma et al. revealed that preeclamptic placentas have more intense sFLT1 staining in the syncytial knots [24]. In another study, Fan et al. showed an increased sFLT1 expression in EVTs in preeclamptic women [11]. However, there is no literature about the expression and distribution of placental sFLT1 in normal pregnancy, especially during early gestation. We showed for the first time that sFLT1 is highly expressed in EVTs and STB in first-trimester placentas, which suggests a physiological role for sFLT1 during early normal pregnancy.

The importance of sFLT1 in normal pregnancy has been emphasized in other studies. For instance, the study by Richard et al. showed high levels of serum sFLT1 in healthy pregnancy compared to a non-pregnant state [10]. Moreover, Kerry et al. found that lower levels of sFLT1 expression are associated with invasive placentation, indicating a critical functional role for sFLT1 in the regulation of placental invasion [25]. sFLT1 has also been suggested to have protective properties in preeclampsia. The first evidence comes from a study by Perucci et al. They showed that sFLT1 knockdown in VEGF overexpressing animals enhances PE-like symptoms in comparison to conditions of only VEGF overexpression [11]. Additionally, Nakakita et al. reported that the removal of sFLT1 in PE has negative consequences, such as the development of fetal bradycardia [26]. More recently, Parchem et al. revealed that reducing PlGF levels has a protective effect against the development of preeclampsia [27]. Taken together, these findings support our hypothesis that the upregulation of sFLT1 plays a protective role during normal and preeclamptic pregnancies.

One possible explanation why sFLT1 is upregulated in EVTs and STB at the maternal–fetal interface could be that both EVTs and STB are in close contact with maternal immune cells; thus, sFLT1 is secreted as an anti-inflammatory cytokine to contribute to immune tolerance. It has been abundantly described in the literature that EVTs and STB are involved in immune tolerance via chemokines and cytokines. For instance, a study by Veras et al. reported that STB has higher levels of programmed death-ligand 1 (PD-L1), which is a immune-suppressive protein, when compared with CTBs in early and term normal placentas [28]. More recently, Michelle et al. summarized that STB expresses several other immune suppressive factors, such as the metabolizing enzyme heme oxygenase-1 (HMOX1) [29]. Furthermore, HLA-G, which is specifically expressed in EVTs, is widely regarded as the pivotal protective factor for a tolerogenic microenvironment at the maternal–fetal interface [30]. Meanwhile, sFLT1 was shown to have an anti-inflammatory function in various immune-related diseases [31,32,33].

To further explore the regulation of sFLT1 production in uncomplicated and preeclamptic pregnancy, we performed in vitro experiments to investigate whether MIF is involved in increased sFLT1 production during differentiation of CTBs into EVTs and STB and whether MIF upregulates sFLT1 expression in a human trophoblast cell line. Moreover, we correlated placental sFLT1 with MIF expression in patients with PE. We found a regulatory relationship between MIF and sFLT1 expression. It was first reported by Perveen et al. that MIF increases sFLT1 expression in a rat model with congenital diaphragmatic hernia [21]. Our results prove the role of MIF in the regulation of sFLT1 expression and show that MIF increases sFLT1 production in early uncomplicated pregnancy and PE. 

The role of MIF in uncomplicated pregnancy and PE has been reported. For example, Krivokuca et al. elaborated that MIF promotes trophoblast migration and invasion [34]. Additionally, the role of MIF in the spiral artery remodeling process was shown by Vilotić et al. [35]. Moreover, a study by Ietta et al. demonstrated that MIF is able to maintain trophoblast homeostasis by preventing abnormal apoptotic death [36]. Furthermore, Hristoskova et al. found that serum MIF concentrations are elevated throughout pregnancy [37], and other studies showed elevated MIF serum levels in PE compared to normal pregnancy [38,39]. Most importantly, Arcuri et al. revealed that MIF can be produced by immune cells and contributes to maternal–fetal immunotolerance via autocrine or paracrine manner [40], which provides a possible inflammation-modulated link between MIF and sFLT1 at the maternal–fetal interface. We confirmed that MIF upregulates sFLT1 expression when CTBs differentiate into EVTs and STB, and that placental MIF positively correlates with sFLT1 expression in PE, which suggests a potential inflammation-associated mechanism between MIF and sFLT1 at the maternal–fetal interface in early uncomplicated pregnancy and PE. 

The regulation of inflammatory factors on sFLT1 expression has been abundantly tested in previous studies. In 2004, Eubank et al. revealed that granulocyte-macrophage colony-stimulating factor (GM-CSF) induces sFLT1 production in human monocytes [16]. More recently, a study by Xia et al. showed that both interleukin-4 (IL-4) and GM-CSF mediate the upregulation of sFLT1 in monocytes [17]. Even more important, tumor necrosis factor-α (TNF-α), agonistic autoantibodies to the angiotensin II type I receptor (AT1-AA), and angiotensin II (ANG II) increase sFLT1 expression during pregnancy in vivo or in vitro [15,41,42]. Taken together, these findings suggest that inflammatory factors serve as an important stimulus for sFLT1 production during pregnancy and firmly support our assumption that sFLT1 plays a potential inflammatory role in pregnancy and PE.

A limitation of our study was that we do not have data on sFLT1 expression in first-trimester placentas or placental explants from women who later go on to develop PE. Currently, there is limited effective prediction for PE, which makes it difficult to obtain placental tissue at early gestation [43].

In conclusion, we reported that sFLT1 has a high expression at the maternal–fetal interface during early pregnancy. Furthermore, we showed that MIF increases sFLT1 expression in early uncomplicated pregnancy and PE, which suggests that sFLT1 is involved in the modulation of inflammation in pregnancy. When it comes to the treatment of PE, negative consequences of sFLT1 removal should be taken into consideration. Further studies should focus on the downstream effect of sFLT1 and its anti-inflammatory mechanisms in uncomplicated pregnancy and PE.

## 4. Materials and Methods

### 4.1. Human Tissue Collection

First-trimester placental tissues were used to investigate sFLT1 and MIF expression. These placentas were also used for isolation of primary cytotrophoblasts as previously described by Eikmans et al. [44]. All samples are from donors (n = 3) who had undergone termination of pregnancy due to social reasons between six and nine weeks gestation.

A term placental cohort, previously documented by van ’t Hof et al. [45], was used to measure *sFLT1* and *MIF* expression. The clinical characteristics are summarized in Table 1. In short, placental tissue specimens were obtained from patients with (n = 14) and without (n = 19) PE, as defined according to the definition by the International Society for the Study of Hypertension in Pregnancy (ISSHP). Cases in which the pregnancy had no preeclampsia, HELLP, preterm birth, decreased birth weight, fetal growth restriction, or infection were included as a normal control group. All placental samples were collected within 24 h of delivery and subsequently embedded in paraffin or frozen at −80 °C.

All samples were collected and handled in accordance with Dutch national ethics guidelines and in accordance with the Code of Conduct regarding the Proper Secondary Use of Human Tissue.

Informed consent was obtained from every patient. The study protocol was approved by the ethics committee of the LUMC with protocol number P16.048 (4 May 2016).

### 4.2. Cell Culture and Differentiation

Primary cytotrophoblasts (CTBs) were cultured and differentiated into EVTs and STB as described before [46]. CTBs were incubated on collagen IV-coated plates in CTB medium, which represents DMEM/F12 supplemented with 0.05 mM 2-mercaptoethanol, 0.2% FBS, 1% penicillin/streptomycin (p/s), 0.3% bovine serum albumin (BSA), 0.5% KnockOut Serum Replacement (KSR), 1% Insulin-Transferrin-Selenium (ITS)-X, 1.5 ug/mL of L-ascorbic acid, 50 ng/mL of epithelial growth factor (EGF), 2 µM CHIR-99021, 0.5 µM A 83-01, 1 µM SB431542, 0.8 mM Valproic acid, and 5 µM Y-27632. For coating of the plates, 5 µg/mL of collagen IV in PBS was incubated for 90 min at 37 °C. After washing with PBS, 0.5 × 10^6^ CTBs were incubated per 2 mL of CTB medium at 37 °C. During culturing, the medium was refreshed every 2–3 days. Cells were transferred to a new coated dish when they had reached a confluence of around 80%.

To study EVTs, CTB cultures were harvested and 0.75 × 10^5^ cells were transferred to a fresh, collagen IV-coated (1 µg/mL) dish and incubated in 2 mL of EVT medium. This medium contained DMEM/F-12 supplemented with 0.1 mM 2-mercaptoethanol, 1% p/s, 0.3% BSA, 1% ITS-X, 7.5 µM A 83-01, 2.5 µM Y-27632, 100 ng/mL of Neuregulin 1 (NRG1), and 4% KSR. At the end of resuspension, Matrigel (final concentration of 2%) was added. After three days, the medium was replaced with the same content but minus NRG1 and with 0.5% Matrigel. At six days, the cells were harvested for further analysis.

To study STB, CTB cultures were harvested and 1 × 10^5^ cells were transferred to a fresh collagen IV-coated (2.5 µg/mL) dish and incubated in 2 mL of STB medium. This medium contained DMEM/F-12 supplemented with 0.1 mM 2-mercaptoethanol, 1% p/s, 0.3% BSA, 1% ITS-X, 2.5 µM Y-27632, 2 µM forskolin (FSK), and 4% KSR. After three days, the medium was replaced with the same content. At six days, the cells were harvested for further measurements.

To study the effect of MIF inhibition on sFLT1 expression, after precoating with appropriate concentration of collagen IV, 0.75 × 10^5^ CTBs per well with EVT medium or 1 × 10^5^ CTBs per well with STB medium were plated in a 6-well plate. After 3 days, medium for EVTs or STBs was changed as described above and 12.5 ug/mL, 25 ug/mL, 50 ug/mL, or 100 ug/mL MIF inhibitor (ISO-1) (Selleckchem) was added. On day 6, all samples were harvested for gene expression analysis using qPCR. As a control, cells were incubated with EVT or STB medium without ISO-1.

To study the effect of MIF on sFLT1, human trophoblast Bewo cells (ATCC) were cultured in DMEM/F12 (Gibco Laboratories, Gaithersburg, MD, USA) supplemented with 10% fetal bovine serum (FBS, Sigma-Aldrich, St. Louis, MO, USA) at 37 °C in 5% CO_2_. To analyze the regulated effect of MIF on sFLT1 production, 3 × 10^5^ Bewo cells per well were plated in a 12-well plate. One day after the Bewo cells reached confluence, they were further cultured in serum-free medium for 24 h and subsequently incubated with 0.2 ng/mL, 2 ng/mL, or 20 ng/mL of MIF (Pepro Tech, Rocky Hill, NJ, USA) for 24 h. As a control, cells were incubated in the medium without MIF. RNA isolation, cDNA synthesis, and qPCR were performed as described below.

### 4.3. Quantitative PCR

To quantify changes in gene expression, total RNA was extracted from frozen placenta sections (10 μm) or cells using TRIzol extraction buffer (ThermoFisher Scientific, Waltham, MA, USA) and converted to cDNA with AMV reverse transcriptase (Roche, Basel, Switzerland) using random hexamer primers. Quantitative real-time PCR was performed using IQ SYBR Green Supermix (Bio-Rad, Hercules, CA, USA) on a Bio-Rad CFX real-time system. Gene expression levels were normalized to the housekeeping gene *GAPDH*. The following primer pairs were used: human GAPDH forward, 5′-CGACCACTTTGTCAAGCTCA-3′ and reverse, 5′ AGGGGTCTACATGGCAACTG-3′; human sFLT1 forward, 5′-CGAGCCTCAGATCACTTGGT-3′ and reverse, 5′-CGATGACGATGGTGACGTT-3′; human MIF forward, 5′-AGCAGCTGGCGCAGG-3′ and reverse, 5′-CTGTAGGAGCGGTTCTGCG-3′.

### 4.4. Immunochemistry

Formalin-fixed placental tissue and cells were embedded in paraffin and sections (4 µm thickness) were cut. Immunochemistry was performed using the following primary antibodies: goat anti-human FLT-1 (1:100, AF321, R&D Systems, Minneapolis, MN, USA), rabbit anti-human MIF (1:200, ABP51791, Abbkine, Wuhan, China), rabbit anti-human Chorionic Gonadotropin (HCGβ, 1:20,000, A0231, Agilent, Santa Clara, CA, US) and mouse anti-human HLA-G (1:800, 11-436-C100, ExBio, Nad Safinou, Vestec, CZ). The appropriate Envision (Dako Cytomation, Glostrup, Denmark) or rabbit anti-goat (Dako Cytomation) horseradish peroxidase-conjugated secondary antibodies were used with diaminobenzidine (Dako Cytomation) as chromogen. Tissue with validated protein expression served as a positive control and an isotype-matched negative control was used for every antibody.

### 4.5. Scoring of Sections

In the biopsy cohort, the MIF-positive area in the placentas was scored semi-quantitatively by two independent observers on images obtained at 100× magnification using the following four categories: MIF-positive area (MIF) very low, MIF low, MIF moderate, and MIF high. Consensus regarding the score of each section was reached and used in the statistical analysis.

### 4.6. Statistical Analysis

Data are expressed as means ± SD. Multigroup comparison was performed using one-way ANOVA followed by the LSD multiple comparison test for subgroup comparison if the overall F-test was significant. To analyze the effect of MIF or ISO-1 stimulation on cells, a linear regression model was used. Independent *t*-tests were performed in healthy and preeclamptic pregnancy groups and Pearson correlation coefficient was determined for correlations using SPSS. The MIF-positive area in preeclamptic placentas and normal controls were compared using a Chi-square test. All normalized gene expression data were log-transformed and an alpha level of 0.05 was used to assess statistical significance.

## Figures and Tables

**Table 1:** Clinical characteristics of controls and preeclampsia cases.

	Control (n = 19)	Preeclampsia (n = 14)
Maternal age (years), mean ± SD	32.2 ± 3.5	31.0 ± 6.7
Gestational age (days), mean ± SD	278.1 ± 6.8	220.2 ± 17.6
Fetal gender (male), number (%)	12 (63.2%)	4 (28.6%)
Birth weight (grams), mean ± SD	3450 ± 488	1435 ± 510
Highest diastole (mmHg), mean ± SD	71.5 ± 8.6 (n = 15)	105.8 ± 8.2 (n = 14)
Proteinuria (present), number (%)	0 (0%) (n = 4)	14 (100%) (n = 14)

## Data Availability

Data sharing not applicable.

## References

[1] Hustin J., Jauniaux E., Schaaps J.P. (1990). Histological study of the materno-embryonic interface in spontaneous abortion. Placenta.

[2] Labarrere C.A., Althabe O.H. (1987). Inadequate maternal vascular response to placentation in pregnancies complicated by preeclampsia and by small-for-gestational-age infants. Br. J. Obstet. Gynaecol..

[3] Farah O., Nguyen C., Tekkatte C., Parast M.M. (2020). Trophoblast lineage-specific differentiation and associated alterations in preeclampsia and fetal growth restriction. Placenta.

[4] Xiao Z., Yan L., Liang X., Wang H. (2020). Progress in deciphering trophoblast cell differentiation during human placentation. Curr. Opin. Cell Biol..

[5] Xu L., Li Y., Sang Y., Li D.J., Du M. (2021). Crosstalk Between Trophoblasts and Decidual Immune Cells: The Cornerstone of Maternal-Fetal Immunotolerance. Front. Immunol..

[6] Fuhler G.M. (2020). The immune system and microbiome in pregnancy. Best Pract. Res. Clin. Gastroenterol..

[7] Perucci L.O., Correa M.D., Dusse L.M., Gomes K.B., Sousa L.P. (2017). Resolution of inflammation pathways in preeclampsia–A narrative review. Immunol. Res..

[8] Maynard S.E., Min J.Y., Merchan J., Lim K.H., Li J., Mondal S., Libermann T.A., Morgan J.P., Sellke F.W., Stillman I.E. (2003). Excess placental soluble fms-like tyrosine kinase 1 (sFlt1) may contribute to endothelial dysfunction, hypertension, and proteinuria in preeclampsia. J. Clin. Investig..

[9] Thadhani R., Kisner T., Hagmann H., Bossung V., Noack S., Schaarschmidt W., Jank A., Kribs A., Cornely O.A., Kreyssig C. (2011). Pilot study of extracorporeal removal of soluble fms-like tyrosine kinase 1 in preeclampsia. Circulation.

[10] Levine R.J., Maynard S.E., Qian C., Lim K.H., England L.J., Yu K.F., Schisterman E.F., Thadhani R., Sachs B.P., Epstein F.H. (2004). Circulating angiogenic factors and the risk of preeclampsia. N. Engl. J. Med..

[11] Fan X., Rai A., Kambham N., Sung J.F., Singh N., Petitt M., Dhal S., Agrawal R., Sutton R.E., Druzin M.L. (2014). Endometrial VEGF induces placental sFLT1 and leads to pregnancy complications. J. Clin. Investig..

[12] Schonthaler H.B., Huggenberger R., Wculek S.K., Detmar M., Wagner E.F. (2009). Systemic anti-VEGF treatment strongly reduces skin inflammation in a mouse model of psoriasis. Proc. Natl. Acad. Sci. USA.

[13] Bus P., Scharpfenecker M., Van Der Wilk P., Wolterbeek R., Bruijn J.A., Baelde H.J. (2017). The VEGF-A inhibitor sFLT-1 improves renal function by reducing endothelial activation and inflammation in a mouse model of type 1 diabetes. Diabetologia.

[14] Nevo O., Soleymanlou N., Wu Y., Xu J., Kingdom J., Many A., Zamudio S. (2006). Increased expression of sFlt-1 in in vivo and in vitro models of human placental hypoxia is mediated by HIF-1. Am. J. Physiol. Regul. Integr. Comp. Physiol..

[15] Parrish M.R., Murphy S.R., Rutland S., Wallace K., Wenzel K., Wallukat G., Keiser S., Ray L.F., Dechend R., Martin J.N. (2010). The effect of immune factors, tumor necrosis factor-alpha, and agonistic autoantibodies to the angiotensin II type I receptor on soluble fms-like tyrosine-1 and soluble endoglin production in response to hypertension during pregnancy. Am. J. Hypertens..

[16] Eubank T.D., Roberts R., Galloway M., Wang Y., Cohn D.E., Marsh C.B. (2004). GM-CSF induces expression of soluble VEGF receptor-1 from human monocytes and inhibits angiogenesis in mice. Immunity.

[17] Xia L., Dong Z., Zhang Y., Zhang X., Song X., Sun M., Hu Y., Liu S., Wang K., Qu X. (2016). Interleukin-4 and granulocyte-macrophage colony-stimulating factor mediates the upregulation of soluble vascular endothelial growth factor receptor-1 in RAW264.7 cells—A process in which p38 mitogen-activated protein kinase signaling has an important role. J. Microbiol. Immunol. Infect..

[18] Harris J., VanPatten S., Deen N.S., Al-Abed Y., Morand E.F. (2019). Rediscovering MIF: New Tricks for an Old Cytokine. Trends Immunol..

[19] Jankauskas S.S., Wong D.W.L., Bucala R., Djudjaj S., Boor P. (2019). Evolving complexity of MIF signaling. Cell Signal.

[20] Krivokuca M.J., Vilotic A., Stefanoska I., Bojic-Trbojevic Z., Vicovac L. (2021). Macrophage migration inhibitory factor in human early pregnancy events and association with placental pathologies. Placenta.

[21] Perveen S., Ayasolla K., Zagloul N., Patel H., Ochani K., Orner D., Benveniste H., Salerno M., Vaska P., Zuo Z. (2019). MIF inhibition enhances pulmonary angiogenesis and lung development in congenital diaphragmatic hernia. Pediatr. Res..

[22] Collier A.R.Y., Zsengeller Z., Pernicone E., Salahuddin S., Khankin E.V., Karumanchi S.A. (2019). Placental sFLT1 is associated with complement activation and syncytiotrophoblast damage in preeclampsia. Hypertens. Pregnancy.

[23] Gaccioli F., Sovio U., Gong S., Cook E., Charnock-Jones D.S., Smith G.C.S. (2023). Increased Placental sFLT1 (Soluble fms-Like Tyrosine Kinase Receptor-1) Drives the Antiangiogenic Profile of Maternal Serum Preceding Preeclampsia but Not Fetal Growth Restriction. Hypertension.

[24] Aletta B., Marlies P., Frans P., Joke S., Jan Antonie B., Augustine R., Kitty B., Ananth K., Hans B. (2013). OP005. Preeclampsia is associated with the presence of transcriptionally active placental fragments in the maternal lung. Pregnancy Hypertens..

[25] McMahon K., Karumanchi S.A., Stillman I.E., Cummings P., Patton D., Easterling T. (2014). Does soluble fms-like tyrosine kinase-1 regulate placental invasion? Insight from the invasive placenta. Am. J. Obstet. Gynecol..

[26] Nakakita B., Mogami H., Kondoh E., Tsukamoto T., Yanagita M., Konishi I. (2015). Case of soluble fms-like tyrosine kinase 1 apheresis in severe pre-eclampsia developed at 15 weeks’ gestation. J. Obstet. Gynaecol. Res..

[27] Parchem J.G., Kanasaki K., Kanasaki M., Sugimoto H., Xie L., Hamano Y., Lee S.B., Gattone V.H., Parry S., Strauss J.F. (2018). Loss of placental growth factor ameliorates maternal hypertension and preeclampsia in mice. J. Clin. Investig..

[28] Veras E., Kurman R.J., Wang T.L., Shih I.M. (2017). PD-L1 Expression in Human Placentas and Gestational Trophoblastic Diseases. Int. J. Gynecol. Pathol..

[29] Williams M.M., Richer J.K. (2022). Revealing Molecular Mechanisms Supporting Trophoblast-Mediated Maternal Immune Tolerance. Endocrinology.

[30] Zhuang B., Shang J., Yao Y. (2021). HLA-G: An Important Mediator of Maternal-Fetal Immune-Tolerance. Front. Immunol..

[31] van Aanhold C.C.L., Bus P., Zandbergen M., Bos M., Berbee J.F.P., Quint K.D., Bruijn J.A., Baelde H.J. (2020). The Vascular Endothelial Growth Factor Inhibitor Soluble FLT-1 Ameliorates Atopic Dermatitis in APOC1 Transgenic Mice. J. Investig. Dermatol..

[32] Seno A., Takeda Y., Matsui M., Okuda A., Nakano T., Nakada Y., Kumazawa T., Nakagawa H., Nishida T., Onoue K. (2016). Suppressed Production of Soluble Fms-Like Tyrosine Kinase-1 Contributes to Myocardial Remodeling and Heart Failure. Hypertension.

[33] Biscetti F., Flex A., Pecorini G., Angelini F., Arena V., Stigliano E., Gremese E., Tolusso B., Ferraccioli G. (2016). The role of high-mobility group box protein 1 in collagen antibody-induced arthritis is dependent on vascular endothelial growth factor. Clin. Exp. Immunol..

[34] Krivokuca M.J., Stefanoska I., Abu Rabi T., Al-Abed Y., Stosic-Grujicic S., Vicovac L. (2015). Pharmacological inhibition of MIF interferes with trophoblast cell migration and invasiveness. Placenta.

[35] Vilotic A., Krivokuca M.J., Stefanoska I., Vrzic Petronijevic S., Petronijevic M., Vicovac L. (2019). Macrophage migration inhibitory factor is involved in endovascular trophoblast cell function in vitro. EXCLI J..

[36] Ietta F., Ferro E.A.V., Bevilacqua E., Benincasa L., Maioli E., Paulesu L. (2018). Role of the Macrophage Migration Inhibitory Factor (MIF) in the survival of first trimester human placenta under induced stress conditions. Sci. Rep..

[37] Hristoskova S., Holzgreve W., Zhong X.Y., Hahn S. (2006). Macrophage migration inhibition factor is elevated in pregnancy, but not to a greater extent in preeclampsia. Arch. Gynecol. Obstet..

[38] Todros T., Bontempo S., Piccoli E., Ietta F., Romagnoli R., Biolcati M., Castellucci M., Paulesu L. (2005). Increased levels of macrophage migration inhibitory factor (MIF) in preeclampsia. Eur. J. Obstet. Gynecol. Reprod. Biol..

[39] Mahmoud S., Nasri H., Nasr A.M., Adam I. (2019). Maternal and umbilical cord blood level of macrophage migration inhibitory factor and insulin like growth factor in Sudanese women with preeclampsia. J. Obstet. Gynaecol..

[40] Arcuri F., Cintorino M., Carducci A., Papa S., Riparbelli M.G., Mangioni S., Di Blasio A.M., Tosi P., Vigano P. (2006). Human decidual natural killer cells as a source and target of macrophage migration inhibitory factor. Reproduction.

[41] Zhou C.C., Ahmad S., Mi T., Xia L., Abbasi S., Hewett P.W., Sun C., Ahmed A., Kellems R.E., Xia Y. (2007). Angiotensin II induces soluble fms-Like tyrosine kinase-1 release via calcineurin signaling pathway in pregnancy. Circ. Res..

[42] Zhou C.C., Ahmad S., Mi T., Abbasi S., Xia L., Day M.C., Ramin S.M., Ahmed A., Kellems R.E., Xia Y. (2008). Autoantibody from women with preeclampsia induces soluble Fms-like tyrosine kinase-1 production via angiotensin type 1 receptor and calcineurin/nuclear factor of activated T-cells signaling. Hypertension.

[43] MacDonald T.M., Walker S.P., Hannan N.J., Tong S., Kaitu’u-Lino T.J. (2022). Clinical tools and biomarkers to predict preeclampsia. EBioMedicine.

[44] Eikmans M., van der Keur C., Anholts J.D.H., Drabbels J.J.M., van Beelen E., de Sousa Lopes S.M.C., van der Hoorn M.L. (2022). Primary Trophoblast Cultures: Characterization of HLA Profiles and Immune Cell Interactions. Front. Immunol..

[45] Van ‘t Hof L.J., Dijkstra K.L., van der Keur C., Eikmans M., Baelde H.J., Bos M., van der Hoorn M.L.P. (2020). Decreased expression of ligands of placental immune checkpoint inhibitors in uncomplicated and preeclamptic oocyte donation pregnancies. J. Reprod. Immunol..

[46] Okae H., Toh H., Sato T., Hiura H., Takahashi S., Shirane K., Kabayama Y., Suyama M., Sasaki H., Arima T. (2018). Derivation of Human Trophoblast Stem Cells. Cell Stem Cell.

